# In-Hospital Predictors of Need for Ventilatory Support and Mortality in Chest Trauma: A Multicenter Retrospective Study

**DOI:** 10.3390/jcm12020714

**Published:** 2023-01-16

**Authors:** Elisa Reitano, Francesco Gavelli, Giacomo Iannantuoni, Silvia Fattori, Chiara Airoldi, Simone Matranga, Stefano Piero Bernardo Cioffi, Silvia Ingala, Francesco Virdis, Martina Rizzo, Nicole Marcomini, Alberto Motta, Andrea Spota, Matteo Maestrone, Roberta Ragozzino, Michele Altomare, Luigi Mario Castello, Francesco Della Corte, Rosanna Vaschetto, Gian Carlo Avanzi, Osvaldo Chiara, Stefania Cimbanassi

**Affiliations:** 1 Department of Translational Medicine, University of Eastern Piedmont, Via Solaroli 17, 28100 Novara, Italy; 2 Emergency Medicine Department, Ospedale Maggiore della Carità, Corso Mazzini 18, 28100 Novara, Italy; 3 General Surgery and Trauma Team, ASST Niguarda, Milano, Piazza Ospedale Maggiore 3, 20162 Milan, Italy; 4 Unit of Medical Statistics and Epidemiology, Ospedale Maggiore della Carità, Corso Mazzini 18, 28100 Novara, Italy; 5 Department of Anesthesiology and Intensive Care, Ospedale Maggiore della Carità, Corso Mazzini 18, 28100 Novara, Italy; 6 Division of Internal Medicine, Azienda Ospedaliera “SS. Antonio e Biagio e Cesare Arrigo”, 15121 Alessandria, Italy; 7 Department of Medical-Surgical Physiopathology and Transplantation, University of Milan, Via Festa del Perdono 7, 20122 Milan, Italy

**Keywords:** trauma, emergency surgery, emergency medicine, chest trauma

## Abstract

Chest trauma management often requires the use of invasive and non-invasive ventilation. To date, only a few studies investigated the predictors of the need for ventilatory support. Data on 1080 patients with chest trauma managed in two different centers were retrospectively analyzed. Univariate and multivariate analyses were performed to identify the predictors of tracheal intubation (TI), non-invasive mechanical ventilation (NIMV), and mortality. Rib fractures (*p* = 0.0001) fracture of the scapula, clavicle, or sternum (*p* = 0.045), hemothorax (*p* = 0.0035) pulmonary contusion (*p* = 0.0241), and a high Injury Severity Score (ISS) (*p* ≤ 0001) emerged as independent predictors of the need of TI. Rib fractures (*p* = 0.0009) hemothorax (*p* = 0.0027), pulmonary contusion (*p* = 0.0160) and a high ISS (*p* = 0.0001) were independent predictors of NIMV. The center of trauma care (*p* = 0.0279), age (*p* < 0.0001) peripheral oxygen saturation in the emergency department (*p* = 0.0010), ISS (*p* < 0.0001), and Revised Trauma Score (RTS) (*p* < 0.0001) were independent predictors of outcome. In conclusion, patients who do not require TI, while mandating ventilatory support with selected types of injuries and severity scores, are more likely to be subjected to NIMV. Trauma team expertise and the level of the trauma center could influence patient outcomes.

## 1. Introduction

Chest trauma represents one of the leading causes of morbidity and the second cause of death in patients with multiple trauma, followed only by head injuries [1]. The correct in-hospital management of chest trauma seems to be of paramount importance to improve patients’ outcomes and decrease the length of stay (LOS) [2]. The proper use of ventilation supports plays a fundamental role in patient management, ensuring the appropriate thoracic expansion and potentially avoiding the development of respiratory complications [3]. Thoracic injuries and the associated pain can impair the proper thoracic expansion, ultimately leading to hypoxemia and acute respiratory failure, and death [4]. The current literature supports the use of non-invasive mechanical ventilation (NIMV) in chest trauma, showing the advantage in terms of decreased LOS and complications development, and improving patient outcomes [5,6,7]. 

Although tracheal intubation (TI) and NIMV are common in clinical practice, the literature on the topic is still insufficient [3,8,9]. Only a few randomized control trials investigated the role of ventilatory support in chest trauma [8,9] and there are no current guidelines on the type of ventilatory support that should be used according to the patient’s severity and the type of injuries.

This study aims to identify the predictors of the need for ventilatory support and the predictors of death in chest trauma patients.

## 2. Materials and Methods

Data about all patients suffering from thoracic trauma admitted to the Niguarda Trauma Center in Milan (Level-One Trauma Center) and the Maggiore della Carità Hospital in Novara (Level-Two Trauma Center) between October 2010 and November 2017 were retrospectively reviewed and analyzed. Data from Niguarda Trauma Center were extracted from the Niguarda Trauma Registry, prospectively updated by a Trauma Team consultant, and annually revised by the Head of the Department. The Trauma Registry of the Niguarda Hospital in Milan was formerly approved for scientific purposes by the Niguarda Milano Area Three Ethics Committee (Record Number 534-102018). Patients of Maggiore della Carità Hospital in Novara were retrospectively extracted from the electronically available records. The study protocol was approved by Maggiore della Carità Hospital Review Board (Record Number 1209/CE).

The study was conducted in conformity with the principles declared to the National Commission for Data Protection and Liberties (CNIL: 2210699) and in accordance with the ethical principles described in the Declaration of Helsinki. Only patients reporting at least one chest injury were included in the study. Demographic data, in-hospital vital parameters [Glasgow come scale (GCS) and peripheral oxygen saturation (SpO2)], type of injuries, mechanism of trauma, need for emergency surgery, chest tube placement, abbreviated injury scale (AIS, 1998 version) of each anatomic region, Injury Severity Score (ISS), Revised Trauma Score (RTS), Probability of Survival (PS) obtained from the TRISS system (TRauma and Injury Severity Score), length of hospitalization (LOS) and in-hospital outcome were evaluated. 

Exclusion criteria were an impaired GCS due to a severe head injury and intubation for agitation on the scene, to avoid bias linked to the intubation due to the neurological deterioration and not to respiratory failure. No patients were transferred to other centers or between centers. 

The American Society of Anesthesiologists (ASA) physical status classification was chosen to summarize comorbidities in an ordinal way. Rib fractures were grouped into four groups: no rib fractures, between 1 and 3, between 4 and 8, and more or equal to 9 rib fractures. Moreover, fractures involving the first and/or second ribs and bilateral rib fractures were considered indicative of a high-energy mechanism of trauma. Any of the following injuries to the chest were also included: pneumothorax, hemothorax, pulmonary contusion, fracture of the scapula, clavicle or sternum, and pleural effusion.

Patients without information on the type of ventilation performed were excluded; the sample was divided into 2 groups based on the type of ventilation to which they were subjected, either invasive ventilation or NIMV. Patients subjected to TI followed by NIMV were considered only in the first group. 

Patients were finally divided into two groups based on the outcome: deceased or survived.

Retrieved data were recorded in a computerized spreadsheet (Microsoft Excel 2016; Microsoft Corporation, Red Mond, WA, USA).

Categorical variables were compared using Pearson’s Chi-square test or Fisher’s test, while numerical variables were studied by *t*-test or Mann-Whitney test according to the distribution of the sample to the Kolmogorov–Smirnov and Shapiro–Wilk tests. A descriptive statistic of the patient population of the two centers involved in the study was provided. 

Multivariate logistic regression was used to provide an odds ratio with 95% confidence intervals, identifying possible predictors of invasive ventilation, non-invasive ventilation, and mortality. The models were adjusted for the center, age, and sex, independently of the statistical significance, and some variables were not included to avoid multicollinearity as expressed below in the text.

Variables with a *p*-value ≤ 0.10 in the univariate model were considered in the multivariable ones. 

Variables with a *p*-value ≤ 0.05 in the multivariate model were considered statistically significant. Based on the characteristics of the multivariate model—which does not allow the inclusion of more than a certain number of variables—and to avoid data repetitions, it was decided not to incorporate some variables in the analysis. The choice of which variables to exclude was based on the multivariable model itself, which due to multicollinearity excluded these variables by itself.

GCS was excluded as it is already represented within the RTS score. The AIS of chest, abdomen, head, and extremities were not included as they are already represented in the ISS score. An ISS > 15 was defined as high [10].

The placement of chest tube thoracostomy and the need for surgery were not considered in the analysis, since PNX often required chest tube placement and surgery always requires intubation. Finally, death probability (TRISS) was excluded from the multivariate model on predictors of deaths, to avoid confounding effects due to the power of these variables on death events. Three different multivariate regression models were performed to identify the independent predictors of TI, NIMV, and death, respectively.

All the analyses were conducted using the statistical software SAS 9.4 (Institute Inc., Cary, NC, USA).

## 3. Results

During the study period, 1080 patients fulfilled the inclusion criteria: 832 (77.4%) chest trauma patients from Niguarda Trauma Center and 248 (22.96%) from Maggiore della Carità Hospital; 845 (78.24%) were male and 235 (21.76%) were female. The median age was 50.0 years [IQR 38.0–63.9]. The age groups were divided according to the previous and most recent WHO classification with a threshold of 65 and 75 years respectively: 815 (75.46%) patients were under 65, 146 (13.52%) between 65 and 75 years, and 119 (11.02%) over 75 years old.

According to the ASA score, 635 (58.91%) patients had an ASA score of one, 359 (33.30%) had an ASA score of two, 79 (7.33%) had an ASA score of three, and 5 (0.46%) patients had an ASA score of four. Data on ASA score was not available in 2 patients (0.18%). 

The mechanism of trauma was represented by motorcycle accidents in 13 cases (29.87%), precipitations in 261 (24.90%), car accidents in 240 (22.90%), pedestrian hits in 142 (13.55%), and bicycle accidents in 75 cases (7.16%). For the other patients, it was not possible to trace the mechanism of trauma. Mechanisms of trauma for patients admitted to both centers are reported in Table 1.

Chest trauma was diagnosed through E-FAST in 868 (80.44%) patients, chest X-ray in 760 (70.44%) patients, and torso-CT in 948 (87.86%) patients. Isolated chest trauma was present in 300 (27.78%) patients, while, in the remaining 780 (72.22%) chest trauma was associated with one or more injuries in another district. Overall, 475 patients (43.98%) showed an abdominal trauma, 639 (59.22%) head injury and 703 (65.09%) reported trauma of the extremities.

According to the different trauma scores, the median chest AIS was 3 [IQR 3–4], the median abdomen AIS was 0 [IQR 0–2], the median head AIS was 1 [IQR 0–3], and the median extremity AIS was 2 [IQR 0–3]. The sample showed a median ISS of 21 [IQR 13–33] and the death probability had a median of 3.78 [IQR 1.10–15.10].

Finally, 402 (37.22%) patients underwent TI, and 219 (22.10%) were treated with NIMV. Ninety-seven patients (8.99%) died. 

Table 1 highlights the principal differences among patients treated in the different centers. Differences in diagnostic imaging and trauma severity between centers were linked to the different trauma center levels, management trauma protocols, and extra-hospital centralization of major trauma between them. 

A comparison between patients subjected or not subjected to TI was performed and reported in Table 2. 

Different variables showed statistical significance in the univariate analysis and were therefore analyzed with multivariate logistic regression. 

The multivariable model confirmed the presence of rib fractures [(*p* = 0.0001, OR: 8.58 (2.57–28.57)], fracture of scapula, clavicle, or sternum [*p* = 0.0458, OR: 1.57 (1.01–2.46)]), hemothorax [*p* = 0.0035, OR: 3.42 (1.50–7.78)], pulmonary contusion [*p* = 0.0241, OR: 1.55 (1.06–2.56)] and a high ISS [*p* = 0.0241, OR: 1.55 (1.06–2.56)] as independent predictors of TI. 

The multivariable model confirmed the presence of rib fractures [*p* = 0.0009, OR: 7.65 (1.43–41.02)], hemothorax [*p* = 0.0027, OR: 4.45 (1.68–11.76)], pulmonary contusion [*p* = 0.0160, OR: 1.83 (1.12–3.00)], and a high ISS [*p* = 0.0001, OR: 1.05 (1.02–1.07)] as independent predictors of need for NIMV (Table 3). 

The multivariate model confirmed the role of the center of trauma care [*p* = 0.0279, OR: 0.40 (0.18–0.91)], age [*p* < 0.0001, OR: 13.28 (6.43–27.45)], peripheral oxygen saturation in the emergency department [*p* = 0.0010, OR: 0.93 (0.89–0.97)], ISS [*p* < 0.0001, OR: 1.08 (0.105–1.10)], and RTS [*p* < 0.0001, OR: 0.79 (0.73–0.86)] as independent predictors of mortality (Table 4). 

## 4. Discussion

This study shows that rib fractures, hemothorax, pulmonary contusions, and a high ISS are independent predictors of both TI and NIMV. The risk of TI and NIMV is increased fourfold if the number of rib fractures is between 4 and 8, and ninefold if 9 or more. On the other hand, the fracture of the scapula, clavicula, or sternum were independent predictors of TI. 

Two different systematic reviews and metanalyses [5,6] showed the superiority of NIMV to both precautionary intubation and high-flow face mask oxygen in patients without contraindications to NIMV. The systematic review and meta-analysis of Duggal A. et al. [7] showed that the early use of NIMV in chest trauma without respiratory distress may prevent TI and complications development. In our study, the same variables were found to be predictors of TI and NIMV. As shown in Table 2, patients subjected to TI showed a higher ISS, RTS, and TRISS, defining a higher overall patient severity. Moreover, fractures of the scapula, clavicula, or sternum were associated with a greater overall patient severity, being a sign of high-energy trauma [11]. 

Table 3 showed that in patients not subjected to invasive ventilation, the same predictors of TI, were also predictors of NIMV. Therefore, patients in whom TI was avoided, but presenting multiple rib fractures, hemothorax, pulmonary contusion, or a higher ISS should undergo NIMV. 

Flagel BT et al. [12,13] showed a correlation between the number of rib fractures and trauma severity, with a baseline mortality rate for patients admitted to the hospital following rib fractures of 10%. The mortality increased up to 40% for patients with more than 6 rib fractures. However, this study did not consider the impact of respiratory support on chest trauma patients’ mortality. In our study, an increased number of rib fractures were associated with an increased need for TI or NIMV as shown in Table 2 and Table 3 but were not independent predictors of mortality.

As shown in Table 4, the type of trauma center, age, and the overall patient severity were independent predictors of mortality. These results are in line with current literature, as different studies showed the protective effect of a Level-one trauma center with a committed trauma team in decreasing mortality rate [14,15,16], while other studies showed an association of death with increasing patient age [2,17,18,19]. Indeed, no specific chest injury was found to be a predictor of mortality, but the overall patient severity seems to be of paramount importance as trauma severity scores such as ISS and RTS were found to be independent predictors of mortality. 

This study presents different limitations, mostly related to its retrospective nature. First, the type of analgesic therapy performed was not considered. It is widely known that the type of analgesic therapy is of paramount importance in chest trauma, as influences the proper thoracic expansion and respiratory exchanges [20,21]. Unfortunately, this information was not available in the Niguarda Trauma Registry and was often missing in the electronic records of Maggiore della Carità Hospital. For the same reason, the timing and setup of NIMV were not available, and therefore could not be reported. 

Finally, as previously stated, the two centers differ in the level of trauma care provided. Niguarda Hospital is a Level-one trauma center with a trained multidisciplinary trauma team, surgical trauma leadership, and dedicated protocols for the management of major trauma patients. 

Maggiore della Carità Hospital is a Level-two trauma center and the management of trauma patients is performed by an emergency physician or by an anesthesiologist, depending on trauma severity, with surgical support available only as required. The different sample size between the centers also reflects the different expertise in trauma management. 

Differences in patients’ characteristics managed in the two different centers are resumed in Table 1. 

Differences among patients’ characteristics are linked to the trauma network disposition in Italy that involves the transport of the most critical patients to the Level-one trauma center [22,23].

Level-one trauma centers have dedicated teams trained in trauma management including minimally invasive treatment of injuries, such as interventional radiology and advanced endoscopy. These resources are not even available in a Level-two trauma center. This explains the higher rates of surgery needed in the Level-two trauma center in comparison to Niguarda Hospital. 

Moreover, as described in Table 1, patients managed at the Level-two trauma center showed a higher rate of respiratory failure despite the lesser overall severity (ISS). The availability of dedicated protocols for trauma management and pain control in the Level-one trauma center probably reduced or prevented respiratory failure in these patients.

However, the outcome variables considered in this study (i.e., TI, and NIMV) are normally carried out in both hospitals and the multivariate analysis was adjusted for confounders to avoid the related bias.

As shown in Table 2 and Table 3, the type of center has no impact on TI and NIMV variables.

However, as the center of treatment was found to be a predictor of mortality, our study underlined the importance of a dedicated trauma team with close cooperation between the different services in trauma management.

Our study identified different variables as independent predictors of the need for ventilatory support. Identified variables seem to be almost the same for TI and NIMV. Therefore, patients with specific injuries and a high ISS not undergoing TI are more likely to be subjected to NIMV. Finally, no specific chest injury was related to mortality, but characteristics of both the trauma center and the patients, as well as the overall trauma severity seem to play an important role. Further prospective and larger studies would be advisable to expand our results and develop clear guidelines on the topic.

## Figures and Tables

**Table 1:** Differences among patients treated in the two centers.

	Niguarda Hospital (Milan) (*n* = 832)	Maggiore Hospital (Novara) (*n* = 248)	Total Patients (*n* = 1080)	*p*-Value
Male [*n* (%)]	653 (78.49)	192 (77.42)	845 (78.24)	0.7210
Age overall [median (IQR)]	49 [38–62]	53.15 [36–67]	38 (63–87)	
Age [*n* (%)]				0.0002
<65 years old	651 (78.25)	164 (66.13)	815 (75.46)	
ears old	95 (11.42)	51 (20.56)	146 (13.52)	
>75 years old	86 (10.34)	33 (13.31)	119 (11.02)	
Median GCS in ED [median (IQR)]	15 [3–15]	15 [15–15]	15 [11–15]	<0.0001
Median SpO2 in ED [median (IQR)]	99 [96–100]	96 [93–99]	98 [96–100]	<0.0001
ASA score [*n* (%)]				0.0092
I	508 (61.13)	127 (51.42)	635 (58.91)	
II	266 (32.01)	93 (37.65)	359 (33.30)	
III	55 (6.62)	24 (9.72)	79 (7.33)	
IV	2 (0.24)	3 (1.21)	5 (0.46)	
Mechanism [*n* (%)]				<0.0001
Car	161 (20.10)	79 (31.89)	240 (22.90)	
Motorcycle	256 (31.96)	57 (23.08)	313 (29.87)	
Pedestrian struck	121 (15.11)	21 (8.50)	142 (13.55)	
Fall	189 (23.60)	72 (29.15)	261 (24.90)	
Cyclist	57 (7.12)	18 (7.29)	75 (7.16)	
Other	17 (2.12)	0	17 (1.62)	
TI [*n* (%)]	336 (40.38)	66 (22.61)	402 (37.22)	<0.0001
TI reason [*n* (%)] *				<0.0001
GCS < 8	246 (73.21)	15 (23.08)	261 (65.09)	
Surgery	34 (10.12)	28 (43.08)	62 (15.46)	
Hemodynamic instability	13 (3.87)	7 (10.77)	20 (4.99)	
Respiratory failure	20 (5.95)	10 (15.38)	30 (7.48)	
Airway obstruction	6 (1.79)	2 (3.08)	8 (2.00)	
Agitation	17 (5.06)	3 (4.62)	20 (4.99)	
E-FAST [*n* (%)]	810 (97.36)	58 (23.48)	868 (80.44)	<0.0001
Chest X-ray [*n* (%)]	691 (83.05)	69 (27.94)	760 (70.44)	<0.0001
Torso CT [*n* (%)]	732 (87.98)	216 (87.45)	948 (87.86)	0.8223
Emergency Surgery [*n* (%)]	313 (37.62)	31 (12.55)	344 (31.88)	<0.0001
Chest AIS’98 [*n* (%)]				<0.0001
<3	145 (17.43)	82 (33.07)	227 (21.02)	
≥3	687 (82.57)	166 (66.94)	853 (78.98)	
Abdomen AIS’98 [*n* (%)]				0.0523
<3	677 (81.37)	215 (86.69)	892 (82.59)	
≥3	155 (18.63)	33 (13.31)	188 (17.41)	
Head AIS’98 [*n* (%)]				≤0.0001
<3	559 (67.27)	209 (84.27)	768 (71.18)	
≥3	272 (32.73)	39 (15.73)	311 (28.82)	
Extremity AIS’98 [*n* (%)]				≤0.0001
<3	577 (69.35)	209 (84.27)	786 (72.78)	
≥3	255 (30.65)	39 (15.73)	294 (27.22)	
Median ISS [median (IQR)]	24 [14–36]	14 [10–22]	21 [13–33]	<0.0001
RTS [median (IQR)]	12 [4–12]	12 [12–12]	12 [10–12]	<0.0001
Death probability TRISS [median (IQR)]	4.80 [1.60–18.70]	1.98 [0.53–4.63]	3.78 [1.10–15.10]	<0.0001
Deceased [*n* (%)]	77 (9.25)	20 (8.10)	97 (8.99)	0.5765

* TI reason was calculated only for subjects who underwent TI. GCS: Glasgow Coma Scale; ASA score: American Society of Anesthesiology score; TI: tracheal intubation; CT: Computed tomography; ED: emergency department; AIS: Abbreviated Injury Scale; ISS: Injury Severity Score; RTS: Revised Trauma Score; TRISS: Trauma Injury Severity Score; IQR: Interquartile range; SpO2: peripheral oxygen saturation; E-FAST: extended focused assessment with sonography for trauma.

**Table 2:** Analysis between patients subjected or not subjected to Tracheal intubation (TI).

				Multivariate Model	
	No-TI (*n* = 678)	TI (*n* = 402)	*p*-Value	OR [95% CI]	*p*-Value
Center [*n* (%)]			<0.0001		0.5365
Niguarda Hospital (Milan)	496 (73.16)	336 (85.58)		0.86 [0.54–1.38]	
Maggiore Hospital (Novara)	182 (26.84)	66 (16.42)		Ref	
Male [*n* (%)]	529 (78.02)	316 (78.61)	0.8223	1.11 [0.71–1.74]	0.6435
Age [*n* (%)]			0.4782		0.3983
<65 years old	504 (74.34)	311 (77.36)		Ref	
65–75 years old	94 (13.86)	52 (12.94)		1.43 [0.85–2.40]	
>75 years old	80 (11.80)	39 (9.70)		1.08 [0.62–1.91]	
Median GCS in ED [median (IQR)]	15 [15–15]	3 [3–14]	<0.0001		
Median SpO2 in ED [median (IQR)	98 [96–100]	99 [95–100]	0.0904		
ASA score [*n* (%)]			0.6684		
I	391 (57.75)	244 (60.85)			
II	232 (34.27)	127 (31.67)			
III	50 (7.39)	29 (7.23)			
IV	4 (0.59)	1 (0.25)			
Mechanism [*n* (%)]			0.0015		
Car	168 (25.49)	72 (18.51)			
Motorcycle	205 (31.11)	108 (27.76)			
Pedestrian struck	83 (12.59)	59 (15.17)			
Fall	139 (21.09)	122 (31.36)			
Cyclist	53 (8.04)	22 (5.66)			
Other	11 (1.67)	6 (1.54)			
Emergency Surgery [*n* (%)]	100 (14.75)	244 (60.85)	<0.0001		
Chest AIS’98 [*n* (%)]			<0.0001		
<3	189 (27.88)	38 (9.45)			
≥3	489 (72.12)	364 (90.55)			
Abdomen AIS’98 [*n* (%)]			<0.0001		
< 3	605 (89.23)	287 (71.39)			
≥ 3	73 (10.77)	115 (28.61)			
Head AIS’98 [*n* (%)]			<0.0001		
<3	587 (86.58)	181 (45.14)			
≥3	91 (13.42)	220 (54.86)			
Extremity AIS’98 [*n* (%)]			<0.0001		
<3	553 (81.56)	233 (57.96)			
≥3	125 (18.44)	169 (42.04)			
1st and/or 2nd rib fractures [*n* (%)]	200 (29.59)	183 (46.10)			
N rib fractures [*n* (%)]			<0.0001		0.0001
0	39 (5.92)	8 (2.07)		Ref	
1–3	308 (46.74)	125 (32.30)		2.51 [0.81–7.72]	
4–8	264 (40.06)	168 (43.41)		3.74 [1.24–11.31]	
>9	48 (7.28)	86 (22.22)		8.58 [2.57–28.57]	
Bilateral rib fractures [*n* (%)]	108 (15.93)	129 (32.17)	<0.0001		
Scapula/clavicle/sternum fractures [*n* (%)]	121 (17.85)	95 (23.63)	0.0216	1.57 [1.01–2.46]	0.0458
Pneumothorax [*n* (%)]	236 (34.81)	188 (46.88)	<0.0001		
Hemothorax [*n* (%)]	24 (3.54)	40 (9.98)	<0.0001	3.42 [1.50–7.78]	0.0035
Pulmonary contusion [*n* (%)]	267 (39.38)	259 (64.59)	<0.0001	1.55 [1.06–2.56]	0.0241
Pleural effusion [*n* (%)]	104 (15.34)	96 (23.94)	0.0004		
Median ISS in ED [median (IQR)]	16 [10–22]	34 [25–42]	<0.0001	1.08 [1.06–1.10]	<0.0001
RTS [median (IQR)]	12 [12–12]	4 [4–12]	<0.0001		
Chest Tube Thoracostomy [*n* (%)]	100 (14.75)	244 (60.85)	<0.0001		
Death probability TRISS [median (IQR)]	2.03 [0.80–4.99]	15.30 [4.26–55.30]	<0.0001		

GCS: Glasgow Coma Scale; ASA score: American Society of Anesthesiology score; TI: tracheal intubation; CT: Computed tomography; AIS: Abbreviated Injury Scale; ISS: Injury Severity Score; RTS: Revised Trauma Score; TRISS: Trauma Injury Severity Score; IQR: Interquartile range; SpO2: peripheral oxygen saturation; ED: emergency department.

**Table 3:** Analysis of patients subjected or not subjected to NIMV.

				Multivariate Model	
	No NIMV (*n* = 546)	NIMV (*n* = 110)	*p*-Value	OR [95% CI]	*p*-Value
Center			0.0227		0.1500
Niguarda Hospital (Milan)	389 (71.25)	90 (81.82)		1.64 [0.84–3.21]	
Novara Hospital (Novara)	157 (28.75)	20 (18.18)		ref	
Male [*n* (%)]	422 (77.29)	92 (83.64)	0.1403	1.39 [0.75–2.59]	0.2945
Age [mean (±SD)]			0.2014		0.0487
<65 years old	416 (76.19)	75 (68.18)		Ref	
65–75 years old	71 (13.00)	20 (18.18)		2.10 [1.12–3.96]	
>75 years old	59 (10.81)	15 (1364)		1.63 [0.80–3.32]	
Median GCS in ED [median (IQR)]	15 (15–15)	15 (15–15)	0.1084		
Median SpO2 [median (IQR)]	98 [96–100]	98 [96–100]	0.4051		
ASA score [*n* (%)]			0.3033		
I	327 (60.00)	57 (51.82)			
II	178 (32.66)	44 (40.00)			
III	36 (6.61)	9 (8.18)			
IV	4 (0.73)	0 (0.00)			
Mechanism [*n* (%)]			0.1257		
Car	138 (25.699)	27 (25.33)			
Motorcycle	166 (31.26)	36 (33.64)			
Pedestrian struck	70 (13.18)	8 (7.48)			
Fall	108 (20.34)	22 (20.56)			
Cyclist	38 (7.16)	14 (13.08)			
Other	11 (2.07)	0 (0.00)			
Emergency Surgery [*n* (%)]	76 (13.92)	20 (18.18)	0.2485		
Chest AIS’98 [*n* (%)]			≤0.0001		
<3	177 (32.42)	9 (8.18)			
≥3	369 (67.58)	101 (91.82)			
Abdomen AIS’98 [*n* (%)]			0.7392		
<3	487 (89.19)	97 (88.18)			
≥3	59 (10.81)	13 (11.82)			
Head AIS’98 [*n* (%)]			0.0079		
<3	483 (88.46)	87 (79.09)			
≥3	63 (11.54)	23 (20.91)			
Extremity AIS’98 [*n* (%)]			0.2725		
<3	451 (82.60)	86 (78.18)			
≥3	95 (17.40)	24 (21.82)			
1st and/or 2nd rib fractures [*n* (%)]	144 (26.37)	49 (45.37)	<0.0001		
N rib fractures [*n* (%)]			<.0001		0.0009
0	36 (6.79)	2 (1.87)		Ref	
1–3	266 (50.19)	33 (30.84)		1.72 [0.34–8.76]	
4–8	205 (38.68)	51 (47.66)		2.81 [0.57–13.88]	
>9	23 (4.34)	21 (19.63)		7.65 [1.43–41.02]	
Bilateral rib fractures [*n* (%)]	73 (13.37)	29 (26.36)	0.0006		
Scapula/clavicle/sternum fractures [*n* (%)]	93 (17.03)	25 (22.73)	0.1560		
Pneumothorax [*n* (%)]	176 (32. 23)	54 (49.09)	0.0007		
Hemothorax [*n* (%)]	13 (2.38)	10 (9.09)	0.0005	4.45 [1.68–11.76]	0.0027
Pulmonary contusion [*n* (%)]	197 (36.08)	63 (57.27)	<0.0001	1.83 [1.12–3.00]	0.0160
Pleural effusion [*n* (%)]	75 (13.74)	25 (22.73)	<0.0001		
ISS [median (IQR)]	14 [10–20]	22 [14–29]	<0.0001	1.05 [1.02–1.07]	0.0001
RTS [median (IQR)]	12 [12–12]	12 [12–12]	0.0874		
Chest Tube Thoracostomy [*n* (%)]	48 (8.79)	29 (26.36)	<0.0001		
Death probability TRISS [median (IQR)]	1.60 (0.70–4.50)	4.50 (1.70–10.50)	<0.0001		

NIMV: Non-invasive mechanical ventilation; GCS: Glasgow Coma Scale; ASA score: American Society of Anesthesiology score; IOT: orotracheal intubation; CT: Computed tomography; AIS: Abbreviated Injury Scale; ISS: Injury Severity Score; RTS: Revised Trauma Score; TRISS: Trauma Injury Severity Score; IQR: Interquartile range; ED: emergency department; SpO2: peripheral oxygen saturation.

**Table 4:** Outcome analysis.

				Multivariate Model	
	Survived (*n* = 982)	Deceased (*n* = 97)	*p*-Value	OR [95% CI]	*p*-Value
Center			0.5765	0.40 [0.18–0.91]	0.0279
Niguarda Hospital (Milan)	755 (76.88)	77(79.38)			
Novara Hospital (Novara)	227 (23.12)	20 (20.62)			
Male [*n* (%)]	772 (78.62)	72 (74.23)	0.3178	1.11 [0.58–2.14]	0.7558
Age [*n* (%)]			<0.0001		<0.0001
<65 years old	765 (77.90)	49 (50.52)		Ref	
65–75 years old	128 (13.03)	18 (18.56)		3.27 [1.49–7.20]	
>75 years old	89 (9.06)	30 (30.93)		13.28 [6.43–27.45]	
Median GCS in ED [median (IQR)]	15 [14–15]	3 [3–13]	<0.0001		
Median SpO2 in ED [median (IQR)	98 (96–100)	97 (90–100)	0.0226	0.93 [0.89–0.97]	0.0010
ASA score [*n* (%)]			0.0058		
I	591 (60.24)	44 (45.36)			
II	321 (32.72)	38 (39.18)			
III	65 (6.63)	14 (14.43)			
IV	4 (0.41)	1 (1.03)			
Mechanism [*n* (%)]			0.0210		
Car	223 (23.40)	17 (17.89)			
Motorcycle	295 (30.95)	18 (18.95)			
Pedestrian struck	128 (13.43)	14 (14.74)			
Fall	226 (23.71)	35 (36.84)			
Cyclist	65 (6.82)	10 (10.53)			
Other	16 (1.98)	1 (1.05)			
Emergency Surgery [*n* (%)]	292 (29.74)	52 (53.61)	<0.0001		
Chest AIS’98 [*n* (%)]			0.0013		
<3	218 (22.20)	8 (8.25)			
≥3	764 (77.80)	89 (91.75)			
Abdomen AIS’98 [*n* (%)]			0.0007		
<3	823 (83.81)	68 (70.10)			
≥3	159 (16.19)	29 (29.90)			
Head AIS’98 [*n* (%)]			<0.0001		
<3	743 (75.66)	25 (26.04)			
≥3	239 (24.34)	71 (73.96)			
Extremity AIS’98 [*n* (%)]			0.0027		
<3	727 (74.03)	58 (59.79)			
≥3	255 (25.97)	39 (40.21)			
1st and/or 2nd rib fractures [*n* (%)]	340 (34.76)	43 (45.26)	0.0414		
N rib fractures [*n* (%)]			0.0050		
0	43 (4.50)	4 (4.40)			
1–3	409 (42.83)	24 (26.37)			
4–8	389 (40.73)	43 (47.25)			
>9	114 (11.94)	20 (21.98)			
Bilateral rib fractures [*n* (%)]	198 (20.16)	39 (40.21)	<0.0001		
Scapula/clavicle/sternum fractures [*n* (%)]	196 (19.96)	20 (20.62)	0.8770		
Pneumothorax [*n* (%)]	383 (39.00)	41 (42.27)	0.5298		
Hemothorax [*n* (%)]	51 (5.19)	13 (13.40)	0.0011		
Pulmonary contusion [*n* (%)]	468 (47.66)	58 (59.79)	0.0225		
Pleural effusion [*n* (%)]	176 (17.92)	24 (24.74)	0.0992		
ISS [median (IQR)]	19 (13–29)	41 (34–54)	<0.0001	1.08 [0.105–1.10]	<0.0001
RTS [median (IQR)]	12 (12–12)	4 (3–11)	<0.0001	0.79 [0.73–0.86]	<0.0001
Chest Tube Thoracostomy [*n* (%)]	193 (19.65)	37 (38.14)	<0.0001		
Death probability TRISS [median (IQR)]	3.20 (1.10–10.25)	77.80 (27.40 96.40)	<0.0001		

GCS: Glasgow Coma Scale; ASA score: American Society of Anesthesiology score; TI: tracheal intubation; CT: Computed tomography; AIS: Abbreviated Injury Scale; ISS: Injury Severity Score; RTS: Revised Trauma Score; TRISS: Trauma Injury Severity Score; IQR: Interquartile range; SpO2: peripheral oxygen saturation; ED: emergency department.

## Data Availability

The data presented in this study are available on request from the corresponding author. The data are not publicly available due to privacy laws.
